# SOCIAL INTEGRATION OF OLDER MEN AND WOMEN ACROSS EUROPEAN WELFARE STATES: ESTABLISHING A NEW COMPARATIVE INDEX

**DOI:** 10.1093/geroni/igae098.0601

**Published:** 2024-12-31

**Authors:** Martina Brandt, Michal Levinsky, Melanie Wagner

**Affiliations:** TU Dortmund, Dortmund, Nordrhein-Westfalen, Germany; HUJI The Paul Baerwald School of Social Work and Social Welfare, Jerusalem, Yerushalayim, Israel; SHARE Berlin Institute, Berlin, Berlin, Germany

## Abstract

Social integration is a crucial factor for health and mortality in old age. However, despite the increasing attention in aging populations, there is currently no instrument available for comparing social integration of older adults across Europe. In this study, we aim to investigate a social integration index established by Berkman and colleagues across welfare regimes and gender in Europe, with a focus on family policy and gender roles. We utilize data from the Survey of Health, Ageing, and Retirement in Europe (SHARE) and conduct multivariate regression analysis to examine the interactions between gender and welfare regimes on social integration based on this index. Our findings suggest that women are less socially integrated than men, and that older adults in more ‘familialistic’ regimes tend to be less socially integrated. Moreover, care regimes are important for the gender gap in social integration. Women tend to be less integrated in family-oriented welfare systems, presumably due to their role as primary caregivers, which can limit their opportunities for social integration activities outside the home. This study contributes to the literature on social integration and highlights the importance of developing policies that promote social integration among individuals, particularly women, in different welfare systems.

